# Cerebellar Heterotopia: Broadening the Neuroradiological Spectrum of KBG Syndrome

**DOI:** 10.1007/s12311-024-01661-6

**Published:** 2024-02-09

**Authors:** Adelaide Carrara, Camilla Mangiarotti, Ludovica Pasca, Davide Politano, Fulvio D.’ Abrusco, Veronica Carmen Barbero, Adriana Carpani, Renato Borgatti, Anna Pichiecchio, Enza Maria Valente, Romina Romaniello

**Affiliations:** 1 Institute for Advanced Study (IUSS), Pavia, Italy; 2 Child Neurology and Psychiatry Unit, IRCCS Mondino Foundation, Pavia, Italy; 3 Department of Brain and Behavior Neuroscience, University of Pavia, Pavia, Italy; 4 Department of Molecular Medicine, University of Pavia, Pavia, Italy; 5 Neuroradiology Department, IRCCS Mondino Foundation, Pavia, Italy; 6 Neurogenetics Research Centre, IRCCS Mondino Foundation, Pavia, Italy

**Keywords:** Cerebellar heterotopia, Cerebral malformation, KBG syndrome, Neuropsychiatric disorder

## Abstract

KBG syndrome is a rare genetic disorder caused by heterozygous pathogenic variants in *ANKRD11*. Affected individuals have developmental delay, short stature, characteristic facial features, and other dysmorphic findings. To date, a spectrum of unspecific neuroradiological defects has been reported in KBG patients, such as cortical defects, white matter abnormalities, corpus callosum, and cerebellar vermis hypoplasia.

Deep clinical and neuroradiological phenotyping and genotype of a patient presenting with mild cognitive and behavioral problems were obtained after written informed consent.

We herein describe the first KBG patient presenting with cerebellar heterotopia, a heterogeneous malformation characterized by the presence of clusters of neurons within the white matter of cerebellar hemispheres.

This novel association broadens the neuroradiological spectrum of KBG syndrome, and further prompts to investigate the potential functions of ANKRD11 in cerebellar development.

## Introduction

KBG syndrome (OMIM, #148,050) is a rare genetic condition characterized by mild to moderate developmental delay, intellectual disability, and distinctive facial dysmorphisms such as a triangular face, bushy eyebrows with possible synophrys, prominent nasal bridge, anteverted nares, abnormal ears, long philtrum, and macrodontia (more frequently of the upper central incisors) [1, 2]. The name is derived from the initials of the first three families in which the condition was characterized (Hermann, 1975) [3]. Other typical associated findings are brachycephaly, short stature, hand and costovertebral malformations, hearing loss, congenital heart defects, cryptorchidism, seizures, and feeding problems [2, 4]. Half of the patients show behavioral and neurodevelopmental disorders such as attention-deficit/hyperactivity disorder (ADHD) and autism spectrum disorder (ASD). A wide and non-specific spectrum of brain malformations has been associated with KBG syndrome, involving supratentorial and posterior fossa structures (i.e., cortical defects, white matter abnormalities, corpus callosum, and cerebellar vermis hypoplasia) [5, 6]. More than 300 affected individuals have been reported in the literature to date [7].

KBG syndrome is genetically determined by heterozygous pathogenic variants in *ANKRD11* (ankyrin repeat domain-containing protein 11) [2], a member of the family of ankyrin repeat-containing cofactors. It is expressed in neurons and glial cells in the developing brain, and it plays a crucial role in brain development by participating in some transcriptional regulatory processes [8, 9]. Loss of function variants (both frameshift and nonsense, mainly involving exon 9) is the most frequent (69%) cause of KBG syndrome, being *ANKRD11* a dosage-sensitive gene [10]. Other underlying genetic mechanisms are intragenic deletions/duplications (14%) as well as copy number variations (CNVs) involving the *ANKRD11* locus (17%) [3, 4, 11]. Missense variants reducing the protein function have been described in the literature as a rarer cause of this condition [12].

In this study, we describe a patient with a clinical and genetic diagnosis of KBG syndrome showing a peculiar neuroradiological pattern of cerebellar heterotopia (CH), which is a congenital malformation classified among disorders of cerebellar foliation.

## Methods

Written informed consent was obtained from the patient’s legal guardians. A clinical, neurological, and dysmorphological evaluation was performed. Moreover, a specific age-related neuropsychological assessment battery was administered.

The patient underwent a 3 T brain MRI with sedation using multiplanar T1- and T2-weighted images with age-appropriate TR and TE values.

Whole exome sequencing (WES) was performed on genomic DNA using Twist Human Core Kit (Twist Bioscience) on a NovaSeq6000 sequencer (Illumina). Reads were aligned to the human reference genome (GRCh37) using BWA v0.7.5 and variants were called using GATK Unified Genotyper. Several in silico tools were employed to predict the pathogenicity of identified variants, including combined annotation-dependent depletion (CADD), Polymorphism Phenotyping v2 (PolyPhen-2), and deleterious annotation of genetic variants using neural networks (DANN). Variants were classified using the American College of Human Society (ACMG) guidelines.

## Case Presentation

**Fig. 1:**
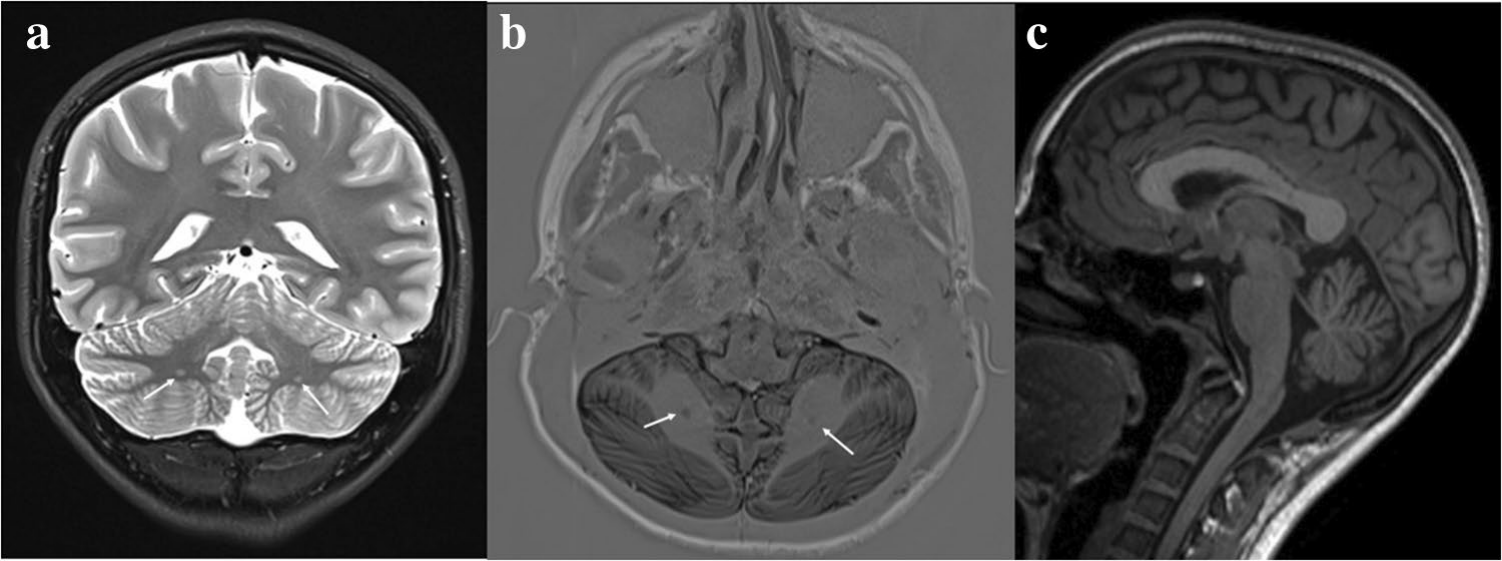
Brain MRI

**Fig. 2:**
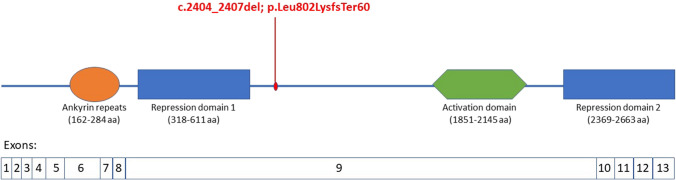
Graphic representation of the ANKRD11 protein and its domains

The patient is a teenager, the first child of unrelated parents, born at term after a physiological pregnancy. The patient presented a length of 49 cm (37th percentile), a weight of 2780 kg (13th percentile), and a head circumference of 32 cm (5th percentile); the Apgar score was within the normal range. Feeding problems were detected from the second year of life, and growth failure with length and weight parameters settling below the third percentile was subsequently observed. Motor developmental milestones were referred to as normally attained. A mild language delay became evident with two-word phrase composition after 2 years of age. During infancy and childhood, some difficulties in school learning were observed, and the serial clinical observations documented mild hypotonia and defective visuo-spatial and fine motor coordination; no cerebellar or other specific neurological signs emerged. Wechsler Intelligence Scale for Children-IV (WISC-IV) was performed at 12 years, showing a cognitive level at the lower normal range (FIQ = 89), with a relative working memory and executive function impairment. Speech and psychomotor rehabilitation associated with special education were implemented. Adaptive functions were characterized by poor daily living skills and socialization. In addition, emotional dysregulation and externalizing behavior were observed. At the same age, a dysmorphological and neurological examination detected facial dysmorphisms characterized by bushy eyebrows and micrognathia and bilateral nystagmus in extreme laterality of gaze. The electroencephalogram did not show epileptiform activity or abnormalities. At brain MRI, two small signal alterations in the subcortical infero-basal cerebellar hemispheres, isointense to gray matter and hyperintense to surrounding myelinated white matter, compatible with small nodules of heterotopia were outlined; moreover, mild vermian cerebellar hypoplasia and thick corpus callosum were present (see Fig. 1). Finally, skull platybasic appearance, hypoplasia of the occipital condyles, and axis dysmorphism were documented. WES detected a pathogenic de novo deletion c.2404_2407del (p.Leu802LysfsTer60) in exon 9 of *ANKRD11*, leading to a diagnosis of KBG syndrome (see Fig. 2).

## Discussion

KBG syndrome is caused by haploinsufficiency of *ANKRD11* [4]. Despite its phenotypic spectrum being wide, our patient presented all the commonest clinical features of KBG syndrome such as developmental delay, neuropsychological and behavioral difficulties, characteristic facial dysmorphisms, history of feeding difficulties, and short stature [3, 7].

However, only a limited number of subjects with KBG syndrome underwent neuroradiological examination, showing unspecific and inconstant supratentorial findings such as cortical malformations (e.g., periventricular nodular heterotopia and focal cortical dysplasia), hippocampal malrotation, corpus callosum hypoplasia, and unspecific white matter defects; in addition, posterior fossa anomalies including Dandy-Walker malformation and cerebellar vermis hypoplasia have also been occasionally reported [3–6]. Brain imaging in our patient showed corpus callosum dysmorphisms and cerebellar vermis hypoplasia which, for the first time in a KBG patient, were associated with a neuroradiological pattern consistent with cerebellar heterotopia. This is a congenital malformation characterized by the presence of clusters of neurons within the white matter of cerebellar hemispheres, and it is classified among disorders of cerebellar foliation, more precisely in the subgroup of rhombomere 1 anomalies [13, 14]. Although its malformative nature suggests a genetic origin responsible of a faulty programming of cerebellar development, no causative genes responsible for CH have been identified to date.

*ANKRD11* gene encodes for a member of the family of ankyrin repeat-containing cofactors, which interacts with p160 nuclear receptor coactivators, inhibits ligand-dependent transcriptional activation, and thus participates in transcriptional regulatory processes [9, 15]. ANKRD11 has a unique protein structure containing two repression domains and one activation domain [8] and it is mainly expressed in neurons and glial cells of the developing brain, playing a crucial role in the proliferative processes of cortical neural precursor cells [8, 9]. It was shown that ANKRD11 regulates pyramidal neuron migration and dendritic differentiation in the developing mouse cerebral cortex, and indeed, its knockdown resulted in delayed radial migration of cortical neurons, significantly reduced branching and dendrite growth, and abnormal dendritic spine morphology [9].

The frameshift variant carried by our patient falls in exon 9 of *ANKRD11*, with exon 9 representing more than 80% of the coding region of the gene [12], and previously reported only in one patient [13]. Genotype–phenotype correlation studies underlined that severe phenotypes are more frequently associated with loss of function variants, while missense variants generally cause milder manifestations of the disease [11]. Interestingly, a statistically significant correlation between short stature and variants falling in exon 9 was reported, as we also observed in our patient [5]. In the Yoda murine model, a heterozygous missense variant in the *ANKRD11* HDAC-binding domain was found to cause craniofacial malformations, altered bone metabolism, and behavioral problems reminding ASD-like anomalies, along with a decreased proliferation of neural precursors with reduced neurogenesis and aberrant neuronal positioning [8]. These studies strengthen the hypothesis that ANKRD11 contributes to the global tuning of transcriptional process in neural precursors, thus playing a role in neurodevelopment and, possibly, cortical development, modulating the expression of other genes. Among these genes, *NCOR2* (nuclear receptor corepressor 2) was reported to be co-expressed in Purkinje cells with *ANKRD11* and *CHD7* (chromodomain helicase DNA-binding protein 7) mutated in CHARGE syndrome (Coloboma, Heart, choanal Atresia, Retardation, Genital, and Ear anomalies) [16], which has also been recently associated with symmetrical heterotopia in the subcortical white matter of bilateral inferior cerebellar hemispheres [17] All these observations, along with the emerging literature linking cerebellar functions to neurodevelopmental disorders such as ASD, prompt further studies to better understand the role of *ANKRD11* gene in cerebellar development.

## Conclusions

This case report broadens the neuroradiological spectrum of KBG syndrome, describing the first KBG patient presenting with bilateral cerebellar heterotopia. We speculate that the presence of this neuroradiological finding is related to the altered functioning of ANKRD11, a protein expressed in the developing brain and cerebellum, leading to a disorganization of brain connectivity and consequently a neurodevelopmental disorder that could hypothetically explain this peculiar phenotype. Therefore, we suggest that future studies should investigate the extent to which *ANKRD11* is expressed at the cerebellar level and the possible impact that its altered functioning would have on clinical manifestations.

The exam was performed at the age of 12 years and 11 months. Coronal T2-TSE (turbo-spin echo) (**a**) and axial IR (inversion recovery) (**b**) images showing two small signal alterations, isointense to gray matter and hyperintense relative to surrounding myelinated white matter, located in the subcortical infero-basal cerebellar hemispheres (arrows), compatible with small nodules of heterotopia. Sagittal T1-weighted image (**c**) showing a thick corpus callosum and a mild inferior vermis hypoplasia in platybasia.

The figure provides a representation of coding exons in scale. The ankyrin repeats are colored in orange (encoded by exons 6–8), the activation domain in green (encoded by exon 9), and the repression domains in blue (encoded by exons 9–13). The identified c.2404_2407del; p.Leu802LysfsTer60 variant is located into the sequence. Since the exon 9 encodes for most of the protein, almost all the other loss of function reported variants are located in this region [10]

## Data Availability

NA.
